# Addressing the Mental and Emotional Health Impacts of COVID-19 on Children and Adolescents: Lessons From HIV/AIDS

**DOI:** 10.3389/fpsyt.2021.589827

**Published:** 2021-06-22

**Authors:** Prerna Banati, Priscilla Idele

**Affiliations:** ^1^UNICEF West and Central Africa Regional Office, Dakar, Senegal; ^2^UNICEF Office of Research at Innocenti, Florence, Italy

**Keywords:** COVID-19, mental health, psychosocial, children, adolescents, HIV/AIDS, rapid review

## Abstract

The COVID-19 pandemic has led to lasting mental health and psychosocial consequences just as were experienced with the HIV epidemic. A rapid review of published systematic reviews on HIV/AIDS and mental health outcomes and responses among children and adolescents was used to identify lessons for the COVID-19 pandemic response. The review found that HIV/AIDS responses to promote mental health, prevent ill-health and treat mental health conditions included diverse interventions at the structural or national, community, household and individual levels. Some of these responses can be easily replicated, others require substantial adaptation, and some can inform development of new innovative offline and online responses to mitigate impact of COVID-19 on mental health of children and adolescents. Programs that mitigate economic impacts including child grants, income generating activities for caregivers, food distribution, health care vouchers, and other economic empowerment interventions can be replicated with minor adjustments. Helplines for vulnerable or abused children and shelters for victims of gender-based violence can be scaled up to respond to the COVID pandemic, with minimal adaptation to adhere to prevention of contagion. Mass media campaigns to combat stigma and discrimination were successfully employed in the HIV response, and similar interventions could be developed and applied in the COVID context. Some programs will need more substantial adjustments. In health facilities, mainstreaming child-sensitive mental health training of frontline workers and task sharing/shifting to community volunteers and social workers as was done for HIV with community health workers, could advance mental illness detection, particularly among abuse victims, but requires adaptation of protocols. At the community and household levels, expansion of parenting programs can help caregivers navigate negative mental health effects on children, however, these are not often operating at scale, nor well-linked to services. Programs requiring innovation include converting adolescent and youth safe physical spaces into virtual spaces particularly for at-risk girls and young women; organizing virtual community support groups, conversations, and developing online resources. Re-opening of schools and introduction of health and hygiene policies, provides another opportunity for innovation - to provide mental health and psychosocial support to all children as a standard package of care and practice.

## Introduction

Evidence of COVID-19 impacts on children and adolescent mental health has been lagging (1). Yet attention to the mental health of children is crucial. The majority of mental health and psychosocial disorders begin during adolescence (10–19 years) and continue into adulthood if not appropriately treated (2), with 10–20% of children and adolescents experiencing diagnosable mental health illnesses (3). Suicide rates are increasing, with young people now being the group at highest risk of suicide in a third of countries, in both developed and developing contexts (2, 4, 5). Depression is the third leading cause of illness and disability among adolescents, and suicide is the third leading cause of death in older adolescents (15–19 years) (2). Apart from mortality and morbidity, mental health and psychosocial illness have other negative consequences for young people, such as lower educational achievements, substance abuse, violence, and poor reproductive and sexual health. If left untreated, it is estimated that mental health disorders which emerge before adulthood can impose a health cost 10 times higher than those that emerge later in life (6).

As the literature of the COVID 19 pandemic grows, we see increasing reports of its mental health consequences on children and adolescents (7–9). However, the literature has been sparse on effective and appropriate responses to mitigate and manage these consequences to protect children and adolescents. This paper seeks to use the expansive evidence generated from the HIV/AIDS pandemic and the impacts on mental and psychosocial well-being of children and adolescents to guide potential responses to COVID-19 impact on children and adolescent mental health. The paper takes a social determinants of health perspective, which recognizes both the direct and indirect impacts of COVID-19 on child mental health. As a result, the analysis covers the direct impact of the disease on the infected person and family; as well as the impact of the pandemic more broadly.

HIV came on the scene about 40 years ago, first considered as a disease of homosexuals, foreigners and sex workers, and shortly after became a generalized epidemic especially in countries across Africa, where the disease has had a disproportionate impact. WHO (10) reports that since the beginning of the HIV epidemic, 75 million people have been infected with the HIV virus and about 32 million people have died of HIV. Globally, 38 million (32.7–44.0 million) people were living with HIV at the end of 2019 (11). Significant mental health and substance use problems have been observed at higher rates among people living with HIV and people who are vulnerable to HIV infection than the general population (12). People with risk factors for HIV and who are vulnerable to mental health conditions often face other significant challenges to accessing and adhering to HIV prevention and treatment modalities. Social stigmas, and criminalization of intersecting risk factors in some contexts (such as sex work, drug use or same-gender sex) present challenges to key populations. These groups experience stigma and discrimination, including incarceration that negatively affect mental health, and this relationship is further compounded by the unfortunate stigma of mental illness in society (13).

The social and behavioral nature of HIV transmission has necessitated investigation beyond the biomedical, to address the social determinants of mental health in HIV affected populations, including addressing risk factors across the human social ecology - from individual, in families and at home, in communities and schools, and in the macro-structural environment. There is growing global evidence that mental disorders in populations – including HIV affected populations - are strongly socially determined, for example, poverty, income inequality, interpersonal violence and forced migration, are key determinants of mental disorders (14).

Evidence from the HIV epidemic successfully shed light on the situation, risk factors and experiences of vulnerable and marginalized individuals who were at heightened risk of co-morbidities. UNAIDS (11) notes the clear positive relationship between HIV prevalence and income disparity; unequal gender norms limiting agency and reduced access to education and economic resources all contribute to HIV risk. The report also notes that young women are at particular risk. In sub-Saharan Africa (SSA) today, women and girls account for 6 out of 10 new infections. Adolescent girls and young women in SSA aged 15–24 account for approximately one quarter of all 2019 infections: disproportionally high given this group constitutes only 10% of the population. Marginalized populations such as commercial sex workers, injecting drug users and people who identify as LGBTI are also at increased risk – and although a small proportion of the population, key populations and their sexual partners carry a disproportionate burden of HIV/AIDS illness.

As we interrogate learnings from HIV on future responses to mental health impacts of COVID-19, it is important to note here that there are both similarities and differences between these two pandemics (15). *Similarities* include the fact thatin both cases, treatment can significantly increase life expectancy and reduce adverse effects of disease progression. Group dynamics and population behaviors play a central role in the spread of both diseases. Stigma and discrimination are prevalent for both diseases, and misinformation is common, with fear driving responses and behaviors. The HIV pandemic has had wide-ranging social and economic consequences, similar to the case of COVID. Commercial sex workers have recently been reported negotiating mask use with their sex partners, with similarities to the HIV experience around condom use (16). *Differences* include the doubling time: early HIV cases doubled over 6–12 months, for SARS-CoV-2 the interval is a matter of days. If left untreated, HIV is mainly life-threatening to all ages while COVID 19 kills a minority of infected individuals, largely older populations and those with existing co-morbidities and vulnerabilities. While a path to ending the COVID-19 pandemic appears closer with the introduction of vaccines, and steep declines in countries with high vaccine rates show this to be possible, an end to the HIV pandemic is much less clear. With treatment, HIV infection is chronic and irreversible while COVID−19 infection is treatable, and an infected person could recover in a short period of time. HIV affects people of reproductive age and can be transmitted from mother to child. Mother to child transmission dynamics of SARS-COV-2 virus are not yet well-understood. Behaviors driving the spread of infection are different - risky sexual behavior and injecting drug use for HIV, while poor hygiene, social and physical proximity are risk factors for COVID-19. Both diseases have re-shaped intergenerational dynamics, however the impacts are manifested differently, with the HIV epidemic leaving behind many orphans to be taken care of by grandparents, while the COVID-19 pandemic has so far had a disproportionate impact on the elderly, although this may vary by country context and demographic structure (17).

## Methodology

We undertook a rapid review of published peer reviewed literature on the impacts of the HIV epidemic on the mental health of children in PubMed, Google Scholar, Cochrane and 3IE databases from June 16 to July 5 2020. We focused solely on published systematic reviews available since 2000. Meta analyses were not included as robust meta analyses are largely statistical procedures that draw from, or are conducted in the context of, a systematic review.

The following search string was used (adapted depending on the search engine employed):

Search string 1: (HIV or AIDS) and (depression or mental or anxiety or suicide or psych^*^) and “systematic review” and (child^*^ or adolescen^*^).

**Figure 1:**
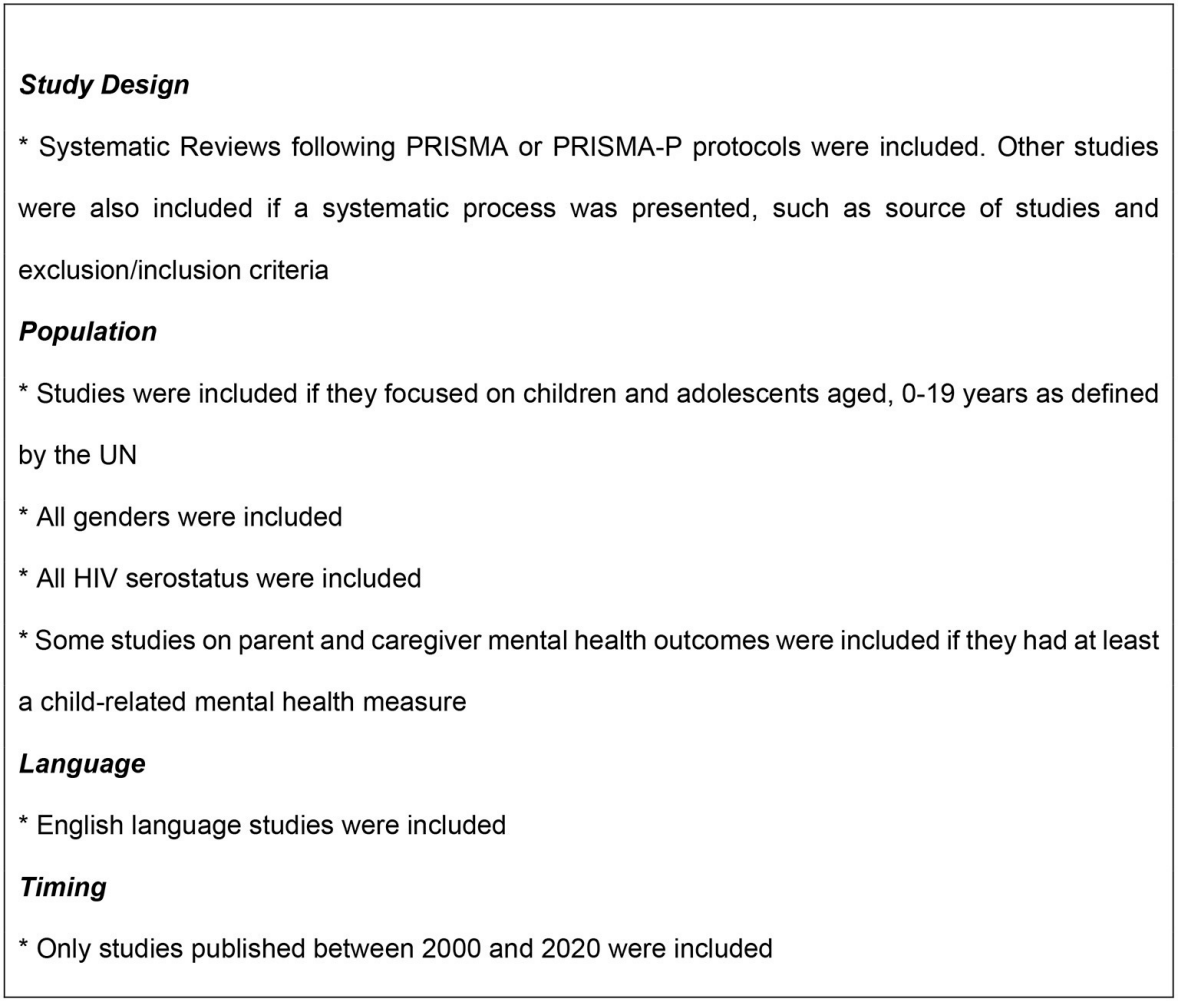
Inclusion criteria for rapid review.

The inclusion criteria are defined in Figure 1. Given the rapid nature of the review, each article was screened and reviewed by only one author. Systematic reviews were analyzed and coded for whether they reviewed determinants, prevalence or mental health disease impact, interventions, pathways or mechanisms of action for mental health outcomes, or mental health diagnostic tools or measures. We coded for geographic setting: low- or middle-income and resource constrained or not. In addition, key evidence gaps identified from each review were summarized.

The authors noted key limitations to the methodology described here. Firstly, the absence of non-English language materials may mean findings are disproportionately focused in anglophone settings. Secondly, publication bias was also noted with the vast majority of articles reporting positive findings, and a small minority reporting negative or inconclusive findings. Due to the nature of the rapid review, only systematic reviews were accessed and only one author summarized. This could bias the evidence accessed and summarized. Finally, the authors noted that systematic reviews are not well-suited to capturing macro-level policy or political shifts or outcomes.

## Data Analysis

The analysis and presentation of data complies with well-accepted methodologies applied in rapid reviews (18). Results from the included publications were synthesized using tables which have provided descriptive details of the publications used in the analysis. The findings provide an overview of the evidence identified, organized thematically. The themes are derived with the goal of providing a sense of the “volume and direction of available evidence” (18).

## Results

**Figure 2:**
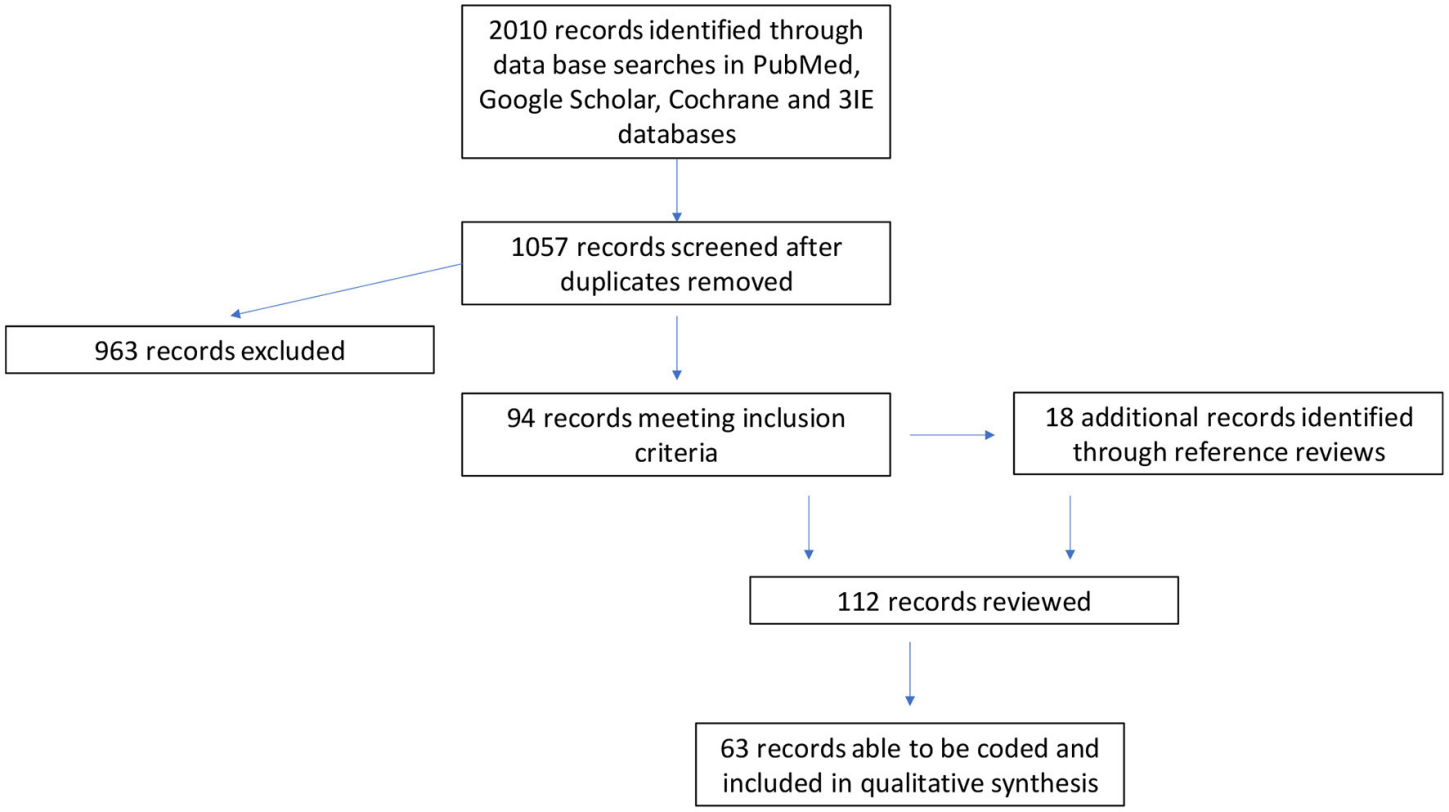
PRISMA flow chart.

Figure 2 provides a flow chart summary of the studies accessed, reviewed and analyzed. Over 2,000 potentially relevant titles resulted in the abstract review of 94 records. 63 systematic reviews were ultimately analyzed. The 63 systematic reviews draw analyses from 2,498 studies. Of the 63 studies reviewed, 38 reviewed determinants, prevalence or mental health disease impact; 32 reviewed interventions with direct or indirect mental health implications; 13 reviewed pathways or mechanisms of action for mental health outcomes, and 7 reviewed mental health or diagnostic tools or measures. Forty-one studies were focused exclusively on HIV affected populations, while the remaining included HIV as one outcome area of interest. Twenty-five studies were geographically focused on low- or middle-income settings. While studies were all used to inform the findings and discussion sections of this paper, 30 focused exclusively on children and adolescents and are summarized in Table 1.

**Table 1:** Summary of systematic reviews focused on children's mental health and HIV.

**Author**	**References**	**Title**	**Number of studies in review**
Govindasamy et al.	(19)	Informing the measurement of well-being among young people living with HIV in sub-Saharan Africa for policy evaluations: a mixed-methods systematic review.	40
Hartog et al.	(20)	Stigma reduction interventions for children and adolescents in low- and middle-income countries: Systematic review of intervention strategies.	61
Toska et al.	(21)	Sex in the shadow of HIV: A systematic review of prevalence, risk factors, and interventions to reduce sexual risk-taking among HIV-positive adolescents and youth in sub-Saharan Africa.	35
Han et al.	(22)	Intergenerational Interventions for People Living with HIV and Their Families: A Systematic Review.	13
Chi and Li	(23)	Impact of Parental HIV/AIDS on Children's Psychological Well-Being: A Systematic Review of Global Literature	30
Qiao et al.	(24)	Disclosure of Parental HIV Infection to Children: A Systematic Review of Global Literature	38
Gourlay et al.	(25)	Barriers and facilitating factors to the uptake of antiretroviral drugs for prevention of mother-to-child transmission of HIV in sub-Saharan Africa: a systematic review.	44
Sherr et al.	(26)	A systematic review examining whether interventions are effective in reducing cognitive delay in children infected and affected with HIV.	17
Sherr et al.	(27)	A Systematic Review of Psychological Functioning of Children Exposed to HIV: Using Evidence to Plan for Tomorrow's HIV Needs.	11
Sherr et al.	(28)	Evidence-based gender findings for children affected by HIV and AIDS - a systematic overview.	11
Haines et al.	(29)	Which HIV-infected youth are at risk of developing depression and what treatments help? A systematic review focusing on Southern Africa.	12
Sherr et al.	(30)	A systematic review of cognitive development and child human immunodeficiency virus infection.	54
Sherr et al.	(31)	Developmental challenges in HIV infected children—An updated systematic review.	21
Rachel et al.	(32)	Disclosure of HIV status to children in resource-limited settings: a systematic review.	32
Spies et al.	(33)	Mental health outcomes in HIV and childhood maltreatment: a systematic review.	34
Kimera et al.	(34)	Challenges and support for quality of life of youths living with HIV/AIDS in schools and larger community in East Africa: a systematic review.	16
Goldberg and Short	(35)	What do we know about children living with HIV-infected or AIDS-ill adults in Sub-Saharan Africa? A systematic review of the literature	47
Skeen et al.	(36)	Interventions to improve psychosocial well-being for children affected by HIV and AIDS: a systematic review.	16
King et al.	(37)	Interventions for improving the psychosocial well-being of children affected by HIV and AIDS.	0
Barry et al.	(38)	A systematic review of the effectiveness of mental health promotion interventions for young people in low- and middle-income countries.	22
Klasen and Crombag	(39)	What works where? A systematic review of child and adolescent mental health interventions for low- and middle-income countries.	134
Sherr and Mueller	(40)	Where is the evidence base? Mental health issues surrounding bereavement and HIV in children.	14
Kuo and Operario	(41)	Caring for AIDS-orphaned children: A systematic review of studies on caregivers.	29
Britto et al.	(42)	Prevalence and correlates of HIV disclosure among children and adolescents in low-and middle-income countries: A systematic review.	22
van Wyhe et al.	(43)	Cross-cultural assessment of HIV-associated cognitive impairment using the Kaufman assessment battery for children: a systematic review.	9
Lloyd and Operario	(44)	HIV Risk among Men Who Have Sex With Men Who Have Experienced Childhood Sexual Abuse: Systematic Review and Meta-Analysis.	12
Medley et al.	(45)	Effectiveness of Peer Education Interventions for HIV Prevention in Developing Countries: A Systematic Review and Meta-Analysis.	31
Krauss et al.	(46)	Disclosure of HIV status to HIV-positive children 12 and under: A systematic cross-national review of implications for health and well-being.	14
Hudelson and Cluver	(47)	Factors associated with adherence to antiretroviral therapy among adolescents living with HIV/AIDS in low- and middle-income countries: a systematic review.	15
McAteer et al.	(48)	A systematic review of measures of HIV/AIDS stigma in paediatric HIV-infected and HIV-affected populations	22

## Findings

Recognizing the complex etiology of child and adolescent mental health and also HIV, we identified six summary findings.

### Poverty and Other Vulnerabilities and Stressors Intersect and Compound Children's Mental Health Risks

Similar to COVID-19, during the HIV pandemic, affected households experienced major shocks to earnings and disruptions to employment resulting in loss of income and precarious financial situations, driving families into hardship and poverty. Parental death left orphans either in the care of other relatives, grandmothers, single parents, or child headed households. Such changing dynamics add stress to already vulnerable households, leading to an increased burden of mental ill health. The vulnerabilities experienced by HIV-infected or affected children are multiple and intersecting and have an impact on their psychological well-being. Findings tended to show that AIDS orphans and vulnerable children had poorer psychological well-being in comparison with children from HIV-free families or children orphaned by other causes (23). HIV-related stressful life events, stigma, and poverty were identified as risk factors, as was pre-existing disability (23).

The vulnerabilities captured are multi-faceted. Socio-demographic variables such as older adolescent girls (29), disability (49) and victims of trafficking were at heightened risk of mental illness (50). Food insecurity was found to drive poor mental health outcomes among HIV affected adolescents and youth (21). Finally, adverse events, such as exposure to abuse and violence, experiences of stigma or discrimination, or taking care of an unwell adult were also detrimental to children's mental health (51, 52). Some evidence is provided that infants, adolescents, children with infected or ill mothers, and children living with severely ill adults are particularly vulnerable (35). A number of factors were found to be protective such as disclosure of HIV status, satisfaction with relationships and social support (29).

We see similar impacts during the COVID pandemic, where extreme financial stress on households is likely to impact families and individuals. Programs that mitigate the economic impacts among the most vulnerable were central to the HIV response and are increasingly being applied to support the COVID−19 response. For HIV, these included child grants, income generating activities for caregivers, cash transfers, food distribution, health care vouchers, community support groups and other economic empowerment interventions. Examples from South Africa, Kenya and Malawi have all shown promising results (53–55). While these responses are being rolled out, the operational mechanisms will need to be adapted to adhere to prevention of contagion (social distancing, wearing masks) which were not needed in the case of HIV. In addition to the individual and household level effects, for COVID-19, we see serious implications in the macro economic and fiscal space. Greater attention to the indirect impacts of shifting patterns of remittances, trade and global financial flows on children's well-being will need further investigation.

### Living Arrangements, Child Abuse and Maltreatment Experienced by AIDS Affected and Infected Children Increase Mental Ill-Health

Similar to COVID-19, the HIV pandemic had a significant impact on social and community fabric, through disruptions in social networks and relationships. During the HIV pandemic, these disruptions were implicated in spikes of child abuse and violence and increases in alternative care arrangements, including at institutions and orphanages and with relatives (56). In our review, risk factors for ill-mental health, particularly depression, include sexual abuse (57), child maltreatment (29) and exposure to violence (58). Spies et al. (33) note that substance abuse, major depressive disorder, and post-traumatic stress disorder were most commonly reported among HIV positive individuals reporting childhood maltreatment. They also noted an association between childhood maltreatment and poor adherence to antiretroviral therapy.

Kuo et al. (41) reviewed the caregiving arrangements for AIDS-orphaned children, identifying findings relevant for COVID-19. Their evidence suggests that caregiving demands are often added to over-burdened and weakened extended family systems particularly in low resource contexts, due to pre-existing financial hardships and reduced social support networks. Their paper also sheds light on the increased psychological and economic stress on caregivers (41).

During the COVID pandemic, undeniably the burden of care is falling on the household, and on women in particular. As women and adolescent girls are largely left responsible for preparation of meals, financial stress in low income settings may mean they could be blamed for insufficient food and subjected to domestic violence. We are currently observing increased reports of levels of abuse, maltreatment and violence, largely documented among women and girls, and often occuring in their own households under conditions of lockdown (59, 60).

During the HIV pandemic, helplines, cash grants for caregivers, accreditation for orphanages, foster care, and adoptions were put in place. For adolescents, safe spaces were created which allowed girls to engage safely with their peers. Expanding the reach of helplines to address child sensitive issues of violence or abuse, shelters, online counseling as well as expansion of virtual safe spaces for example, could offer an opportunity for the COVID-19 response. Mainstreaming mental health training of frontline COVID-19 health and community workers to detect mental distress, particularly among victims of violence, can be an effective approach.

### The Functioning and Quality of Health Systems Have Impacts on Mental Health Outcomes of Children

Many have expressed frustration with long-standing capacity gaps, poor service accessibility and inadequate mental health policy provisions in low- and middle-income countries, critical components required to ensure an adequate health system that delivers good mental health for all (61). However, few studies in our review sought innovations to address the shortfalls. Example approaches identified include: task sharing of mental health care which was observed to improve the reach and effectiveness of services in rural and other low-resource settings; lay health workers to deliver care and community-based packages across outcomes and social support services to pregnant women at risk was seen to be effective, home based care; and non-specialist providers in mental health and neurology and physician-nurse substitution were also identified as possible approaches.

A number of studies noted the need to embed mental health services within the primary health care response, yet this approach was not assessed in any study we reviewed. Integrated models, such as adding a service to an existing service, and those that work both at community and in facilities have been tried successfully (38) but more research is needed on how to make this work effectively. Community workers and volunteers have played a key role in supporting HIV infected patients. They have also assisted in task shifting and task sharing in roles that could easily be done by a lay person such as rapid testing, supply of medicine, follow up for adherence and referrals. In Zambia – adherence support extension workers were recruited, in Malawi they took the shape of community volunteers and patients receiving ART, and South Africa has had very successful community workers models (62, 63). Of relevance to COVID-19, in a systematic review by Mwai et al. community health workers expanded the HIV response by successfully providing patient support (such as counseling, home-based care, education and livelihood support), and health service support (such as screening, referrals and surveillance). Potentially, detection and referrals to mental health services could be layered onto existing community health worker models, though this would require adaptation for the COVID-19 context, including revised protocols and additional protective equipment.

### School-Based Platforms Can Provide Effective Mental Health and Psychosocial Support at Scale

While there were differential effects for gender and age, classroom-based interventions – particularly for adolescents - have reduced symptoms of common mental disorders (38). In the review by (38) thirteen mental health promotion and universal prevention interventions implemented schools were explored for their potential to improve mental health outcomes. The authors noted the interventions spanned across the development of social, emotional, problem solving and coping skills, to mental health promotion combined with physical fitness programs, stress reduction, sexuality education programs, and including art interventions and peer support interventions led by teachers. Two interventions were designed specifically to support AIDS orphaned children. After-school recreational activities had a significant positive impact on children and adolescents' externalizing and internalizing problem scores and also improved parental support as a result of parental involvement in the structured activities. Classroom based interventions were reported to have a significant positive effect on both children and adolescents in terms of improved social and emotional well-being, communication skills and reduced conduct and peer problems and hyperactivity levels. Specific gender effects were reported in one classroom based intervention where significant reductions in psychological difficulties and aggression were seen in males only and improved prosocial behavior among females only. All the universal life skills and resilience school-based interventions identified reported significant positive effects on students' mental health and well-being in terms of improved self-esteem, motivation, and self-efficacy (38) and these interventions, if delivered online, would also be relevant for children experiencing COVID-19 lockdowns. Evidence was mixed for peer education programs in developing countries. Some studies suggested these are moderately effective at improving knowledge and some selected behavioral outcomes but show no significant impact on biological outcomes (45).

There were also promising findings among multicomponent interventions in school and community settings (38). There is evidence that school-based health centers are popular with young people and provide important mental and reproductive health services. Gaps in school-based programming was also evident, as the preponderance of such programs addressed older children, and younger children in LMIC primary schools were often not subject of study. The authors report greater effectiveness of multi-component interventions, which they identify as ‘'interventions that adopt a social competence approach and develop supportive environments,” in comparison to interventions that narrow in on specific problem behaviors. The review notes the efficacy of whole school approaches, which have recently been evaluated by RCTs in Bihar and the UK and shown to be effective in reducing externalizing behaviors and bullying (64).

By early March, COVID-19 had resulted in the closure of schools in over 108 countries (65). As schools begin to slowly reopen, teachers and administrators will be at the frontlines, and play an important role in detecting and mitigating mental health impacts of adverse experiences of lockdown, stigma, social isolation and experience and exposure to disease and deaths. However, they cannot do this alone. As schools introduce new health and hygiene policies, they need to be mindful of mental health implications and include these as a standard part of their health and hygiene protocols.

### Positive Coping, Relationships With Caregivers, Family and Social Support Can Improve Child Mental Health Outcomes

The evidence confirms that individual coping skills, trusting relationship with caregivers and social support protected HIV-affected children against negative mental health effects, with some evidence that these were most impactful among those most stressed (23). Adaptive coping and social support were considered critical to overcome the structural and economic barriers associated with poverty that led to mental ill-health (19) and would be relevant for children experiencing poverty and economic stress as a result of financial crises borne of the COVID-19 pandemic.

There was little reflection of the quality and content of caregiving provided nor information on the challenges of caring for children orphaned by AIDS or effects on caregivers' health and well-being (41). However, a few of the reviews engaged with parenting – noting parent training is a highly promising intervention for behavioral disorders and can bring down rates of conduct problems significantly (39). Evidence suggests that parenting interventions appear to be at least as effective when implemented in countries that are culturally different than those in which they were developed. Gardner et al. (66) report that extensive adaptation was not necessary for the successful transportation of these types of interventions (66).

Applying these lessons today, expanded parenting programs which help caregivers navigate the negative mental health effects on children, may help to mitigate the long-term impacts of COVID-19 (67, 68). An open-access hub of online resources on parenting during COVID-19 has been launched by a consortium of agencies and academics (69). Wide use of such resources – which focus on building positive relationships, diverting and managing bad behavior, and managing parenting stress – can help reduce caregiver and parental stress and strengthen child-parent bonds and build stronger relationships between children and their caregivers.

Our review shows that unintentional or forced HIV disclosures were common (24). In LMIC settings, full disclosure of HIV status among children and adolescents living with HIV was uncommon (42). Findings regarding the impacts of disclosure were mixed but disclosure tended to have long-term positive impacts on the well-being of children, parents and family in general (24). Within the COVID-19 context, disclosure to family has not been as structured as seen during the HIV experience, which has well-established protocols for counseling and testing, complemented with guidelines for disclosure. For example, WHO provides evidence-based guidelines to health workers providing disclosure counseling for children under the age of 12, in recognition of their ‘'emotional and aptitudinal ability to understand and cope with the nature of the illness, stigma, family relations and concerns about social support” (70). Increasing concerns can be seen around privacy of COVID19 testing, with documented instances of people being shunned and stigmatized emerging (71). Lessons from HIV suggest that COVID-19 disclosure should be sensitive to the child's age and perceived ability to understand the meaning of infection (32). Caregivers may need support from health care providers during the disclosure process. Evidence-based guidelines incorporating the developmental status of the child and adapted to cultural context, taking account of caregiver perceptions are prerequisite to enhancing disclosure in local contexts (42). Development and dissemination of such guidelines for COVID-19 disclosure, accompanied by health-worker training and counseling, would be a useful contribution.

Family support should form part of mental health prevention strategies (72). Such support needs to embrace a number of sectors such as housing, education, and employment which need to work together with support from voluntary and mental health sectors. Mobilization of local communities – such as was done for HIV – needs to be undertaken, and may include community outreach and working with community leaders to ensure inclusion of mental and physical health concerns in COVID-19 programs. In response to COVID-19, community groups have been successful at organizing virtual support groups, community conversations (72), developing online resources and even joint singing events (73). Sustainable financial and capacity development support to community ventures can help foster resiliency and build back better in anticipation of future outbreaks.

### Stigma and Discrimination Can Have Significant Impacts on Children's Emotional Lives

As seen during the COVID-19 pandemic and the HIV epidemic, experiences of stigma have had a wide range of negative outcomes for children (34, 74). In a study focusing on HIV treatment adherence, the authors note that interventions to reduce stigma should target multiple levels of influence - intrapersonal, interpersonal and structural - in order to have maximum effectiveness (75). Findings suggest that children and adolescents are under-represented in stigma reduction interventions in low- and middle-income settings (20), suggesting more stigma reduction interventions are needed that address a wider variety of stigmas, with children as direct and indirect target group.

Increasingly, we see emerging stigmatization of COVID-19 patients or their families, creating situations that are not mentally or psychologically healthy and are likely to cause much stress, anxiety or depression in the long run. Such experiences can instill a sense of fear and degenerate into loneliness, social and emotional isolation. Community, religious and faith-based organizations have had a positive role in combatting HIV stigma and discrimination in sub-Saharan African (76). While not many were well-measured, mass media campaigns were developed to respond to the HIV pandemic that proved to be effective at reducing stigma (77), and similar interventions could be developed and applied in the COVID-19 context.

## Discussion

Policymakers and practitioners seeking actionable results to manage the mental health consequences of COVID-19 can benefit from the experiences of HIV. The analysis presented in this paper can be categorized into three types of responses: those that can be easily replicated, those require substantial adaptation, and those that require development of new innovative offline and online responses to mitigate negative consequences of COVID-19 on mental health of children. A summary is presented in Table 2. Many of the programs that mitigate economic impacts including child grants, income generating activities for caregivers, cash transfers, food distribution, health care vouchers, community support groups and other economic empowerment interventions can be replicated with minor adjustments. Given multiple vulnerabilities compound risk of poor mental health in children, cash programs, for example, should expand to include multi-dimensionally poor children and their families. Evidence shows that cash programs have some success in reducing mental ill health (51). Ideally, these programs include “plus” components that link households receiving cash to mental health services.

**Table 2:** Summary of COVID-relevant responses for children's mental health derived from HIV/AIDS rapid review.

**Ease of application to COVID**	**Easily replicated**	**Requires adaptation**	**Requires innovation**
**Platform**			
Individual	• Helplines for psychosocial support. • Counseling services. • Shelters for victims of gender-based violence.	• Peer programs linked to provision of services.	• Converting adolescent and youth safe physical spaces into virtual spaces.
Household	• Child grants. • Income generating activities for caregivers. • Cash transfers and cash-plus programs. • Food distribution. • Health care vouchers. • Economic empowerment interventions.	• Expansion of parenting programs.	
Community	• Community support groups. • Mass media campaigns to combat stigma and discrimination were successfully.	• Community mobilization initiatives. • Community outreach workers and programs with community leaders. • Mental health clinics in every school that re-opens, and equipping them with trained service providers, robust protocols and referral pathways.	• Virtual community support groups and social entertainment programs. • Online resources including modules devoted to positive mental health for students as part of on-line curricula. • Introduction of stringent health and hygiene policies during and after reopening of schools. • Standard package of care and practice in all schools upon their reopening.
Health System		• Mainstreaming child-sensitive mental health training of frontline health workers. • Task shifting and task sharing with Community Health Workers.	

Helplines for psychosocial support to vulnerable or abused children and counseling services and shelters for victims of gender-based violence can be scaled up to respond to the COVID-19 pandemic, with some minimal adaptation to adhere to prevention of contagion. Mass media campaigns to combat stigma and discrimination were successfully employed in the HIV response, and similar interventions could be developed and applied in the COVID-19 context.

Some programs will need more substantial adjustments. At the health facility level, mainstreaming child-sensitive mental health training of frontline health workers and task sharing and task shifting to community volunteers and social workers as was done for HIV with community health workers, could advance detection of mental illnesses, particularly among victims of abuse, but would require adaptation of protocols. At the community and household levels, expansion of parenting programs can help caregivers navigate the negative mental health effects on children, however, these are not often operating at scale, nor with adequate linkages to community-level services. Community mobilization initiatives were commonly seen during the HIV pandemic, as were community outreach workers and programs with community leaders. However, such community-based initiatives will need substantial adaptation and re-orientation given the closure of many community spaces and schools, as well as the dissolution of community ties as a result of home-isolation and lockdown. The school platform can also be harnessed for mental health service delivery particularly in low income contexts, where schools have not been exploited for child mental health delivery. Adding mental health clinics into every school that re-opens, and equipping them with trained service providers, robust protocols and referral pathways can ensure every child can benefit. With the reopening of community venues and schools, new community groups can also be created to advance community mobilization for children's mental health, but this will require adaptation and planning.

Programs requiring innovation include converting adolescent and youth safe physical spaces into virtual spaces particularly for at-risk girls and young women. There have been some successful pilots for violence prevention during COVID-19 (78). Innovating similar models for mental health among young people could be advanced. Expanding virtual community support groups, social entertainment, and developing online resources including modules devoted to positive mental health for students as part of on-line curricula could be an effective approach. Re-opening of schools and introduction of stringent health and hygiene policies, provides another opportunity for innovation - to provide mental health and psychosocial support to all children as a standard package of care and practice.

The review also provided an opportunity to assess evidence gaps. The majority of reviews acknowledged the need for better measurement, research and data on HIV and children's mental health, identifying important gaps that are worthwhile to mention here and important to keep in mind as the evidence base for COVID-19 evolves.

One of the largest gaps noted was the need for specific data on vulnerable populations such as women, children and young people, heterosexual men, drug users and trafficked individuals. During the early parts of the HIV pandemic, age and sex disaggregated data were not available, excluding information on children. Compared to adults, this resulted in delays in the prevention, detection and treatment of affected and infected children. Once age and sex-disaggregated data were made available, children were understood to be directly affected by HIV. Work by Idele et al. reminds us that we should not make this same mistake for COVID-19 (17).

Our review of evidence from HIV exposed the absence of gendered data and analysis. One systematic review noted that of the 15 studies on bereavement they included, seven analyzed data by gender with mixed findings (28). The review noted that major policies fail to provide gender data for young children, despite scientifically established gendered mental health impacts manifesting in adulthood.

Another gap commonly cited was the need for more quality research empirically examining causal pathways or mechanisms of action (33, 35). The absence of such evidence has hampered detection of entry points for action and limited the development of effective interventions. Future COVID research would benefit from more attention to causal inference and further characterization of processes and circumstances, as well as variation, related to vulnerability and resilience.

Long term consequences will also need to be monitored and longitudinal designs and rigorous developmentally-sensitive measurement is needed to inform the design of large-scale policies and programming for children, adolescents and youth (35). Importantly, a number of studies reported the need for intergenerational evidence, both on experiences and interventions on the mental health of children and their families (22).

Importantly, the methodology prevented us from unpacking the role of national leadership and effective public policy in the promotion, prevention, detection and care of mental health conditions in children. Our experience of HIV responses in sub-Saharan Africa suggests that effective national leadership made a significant difference by shifting discourses, combatting stigma and expanding prevention, treatment and care through decentralized approaches, including in Kenya, Uganda and Tanzania. To systematize these, robust policy analysis, which is not often seen in the peer reviewed literature, is needed.

**Figure 3:**
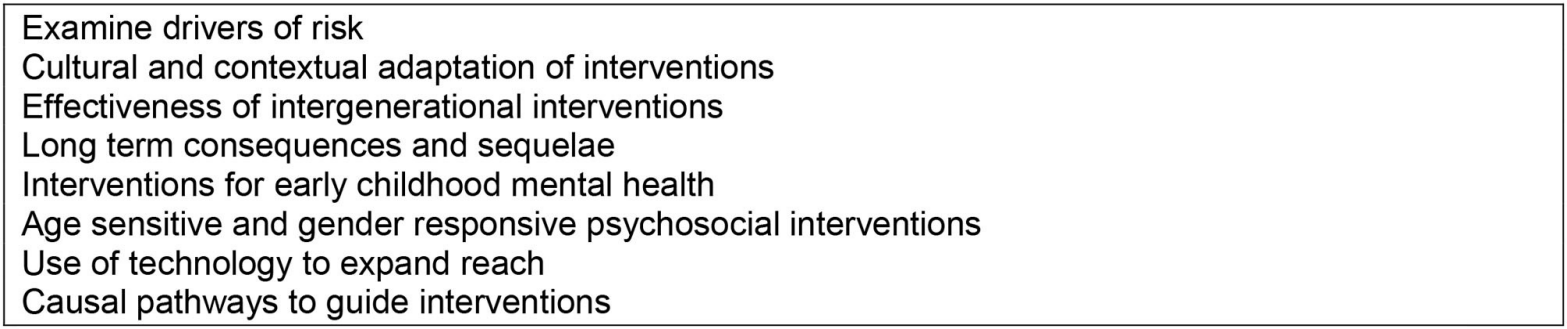
Children's mental health evidence gaps identified from Rapid Review of HIV/AIDS literature.

Figure 3 describes other research gaps that were identified as a result of our review. It is possible that the absence of mental health data, analysis and research reflects the low priority it has in many countries. With the growing momentum to institutionalize and embed mental health into our public health and social systems, we hope these trends will soon reverse.

## Data Availability Statement

The original contributions presented in the study are included in the article/supplementary material, further inquiries can be directed to the corresponding author.

## Author Contributions

PB and PI conceptualized paper and shared writing. PB undertook analysis for rapid reviews. All authors contributed to the article and approved the submitted version.

## Conflict of Interest

The authors declare that the research was conducted in the absence of any commercial or financial relationships that could be construed as a potential conflict of interest.
